# Trends in Pulmonary Tuberculosis Mortality: A Population-Based Study in a Northern Vietnamese Province, 2005–2008 and 2011–2018

**DOI:** 10.3390/tropicalmed11040099

**Published:** 2026-04-10

**Authors:** Ngoan Tran Le, Ngan Dieu Thi Ta, Quyet Quang Nguyen, Thanh C. Bui, Joshua T. Mattila, Suresh V. Kuchipudi, Toan Ha

**Affiliations:** 1 Department of Health Management, Nguyen Tat Thanh University, Ho Chi Minh City 700000, Vietnam; 2 Department of Infectious Diseases, Hanoi Medical University, Hanoi 100000, Vietnam; tadieungan@hmu.edu.vn; 3 CDC Lang Son, Lang Son City 240000, Vietnam; quyetthang689@gmail.com; 4 Department of Family and Preventive Medicine, College of Medicine, University of Oklahoma Health Campus, Oklahoma City, OK 73104, USA; thanh-c-bui@ouhsc.edu; 5 TSET Health Promotion Research Center, Stephenson Cancer Center, University of Oklahoma Health Sciences Center, Oklahoma City, OK 73104, USA; 6 Department of Infectious Diseases and Microbiology, School of Public Health, University of Pittsburgh, Pittsburgh, PA 15213, USA; jmattila@pitt.edu (J.T.M.); skuchipudi@pitt.edu (S.V.K.); 7 Center for Vaccine Research, School of Medicine, University of Pittsburgh, Pittsburgh, PA 15213, USA; 8 Department of Microbiology and Molecular Genetics, School of Medicine, University of Pittsburgh, Pittsburgh, PA 15213, USA; 9 Department of Public Health Sciences, School of Medicine, University of Connecticut, Farmington, CT 06030, USA

**Keywords:** tuberculosis, pulmonary tuberculosis, gender disparities, age-standardized mortality rate, population-based mortality study, Lang Son Province, Vietnam

## Abstract

Tuberculosis (TB) remains a major public health burden in Vietnam, yet few studies have examined pulmonary TB mortality trends at sub-national levels, where local epidemiological patterns may differ substantially from national averages and reveal high-risk populations requiring targeted interventions and inform resource allocation. Lang Son, Vietnam, is a mountainous border province with many ethnic minority residents, and extensive cross-border movement creates distinct challenges for TB surveillance and treatment adherence. Although mortality has declined in line with national trends, rates in this border province remain higher than those in Vietnam’s major urban centers. This disparity suggests a hidden burden where Lang Son’s unique geographic challenges and ethnic diversity create health inequities that are often obscured by favorable national-level averages. To better understand local epidemiological patterns, this study examined temporal trends and gender differences in pulmonary TB mortality in Lang Son Province over a 12-year period (2005–2008 and 2011–2018). Using data from a population-based mortality registration system, we calculated crude and age-standardized mortality rates (ASR) per 100,000 person-years. Temporal trends were assessed using Poisson regression. The overall ASR was 7.7 per 100,000 person-years among men (95% CI: 6.5–9.0) and 1.9 among women (95% CI: 1.3–2.7), yielding a male-to-female ASR ratio of 4.1. The age-standardized pulmonary TB mortality declined by approximately 49.2% (from 6.3 (95% CI: 4.1–9.2) to 3.2 (95% CI: 1.9–4.9) per 100,000 person-years; *p* = 0.025). Notably, 69.9% of deaths occurred in individuals under age 70. While declines were observed in both sexes, sex-specific temporal trends were not statistically significant (*p* > 0.05). Despite these improvements, persistently higher mortality among men and older adults highlights ongoing inequities in TB outcomes within the province. These pre-pandemic findings provide an essential epidemiological baseline for assessing COVID-19’s impact on TB control and underscore the need for age- and gender-targeted interventions at sub-national levels to accelerate Vietnam’s progress toward TB elimination.

## 1. Introduction

Tuberculosis (TB) remains a significant public health challenge globally and in Vietnam. Globally, in 2024, an estimated 10.7 million people newly acquired TB, making TB the leading infectious cause of death worldwide, and it remains the leading infectious cause of death [1,2]. The WHO’s End TB Strategy remains a key priority to address this global burden [3].

Vietnam ranks among the 30 countries with the highest TB burden globally, placing 12th overall and 10th for drug-resistant TB cases [1]. Since the formation of the National TB Program (NTP) in 1986, the country has undergone decades of programmatic evolution. During the directly observed treatment, short-course (DOTS) era (1986–2000), Vietnam successfully expanded the WHO-recommended DOTS strategy, achieving near-universal district coverage and maintaining cure rates above 85% [4,5]. The National Priority Phase (2000–2020) marked TB as a national health priority, contributing to a substantial decline in mortality, from 28 per 100,000 in 2005 to approximately 11 per 100,000 in 2024 [6,7]. Most recently, the Digital and Strategic Modernization Phase (2021–present) has emphasized active case-finding and management of drug-resistant TB through the National Strategic Plan (2021–2025) and the Vietnam TB Information Management Electronic Surveillance (VITIMES) system, highlighting Vietnam’s continued commitment to TB control [8] and reaching the target set by the National Action Plan to End TB by 2030 [9].

**Figure 1:**
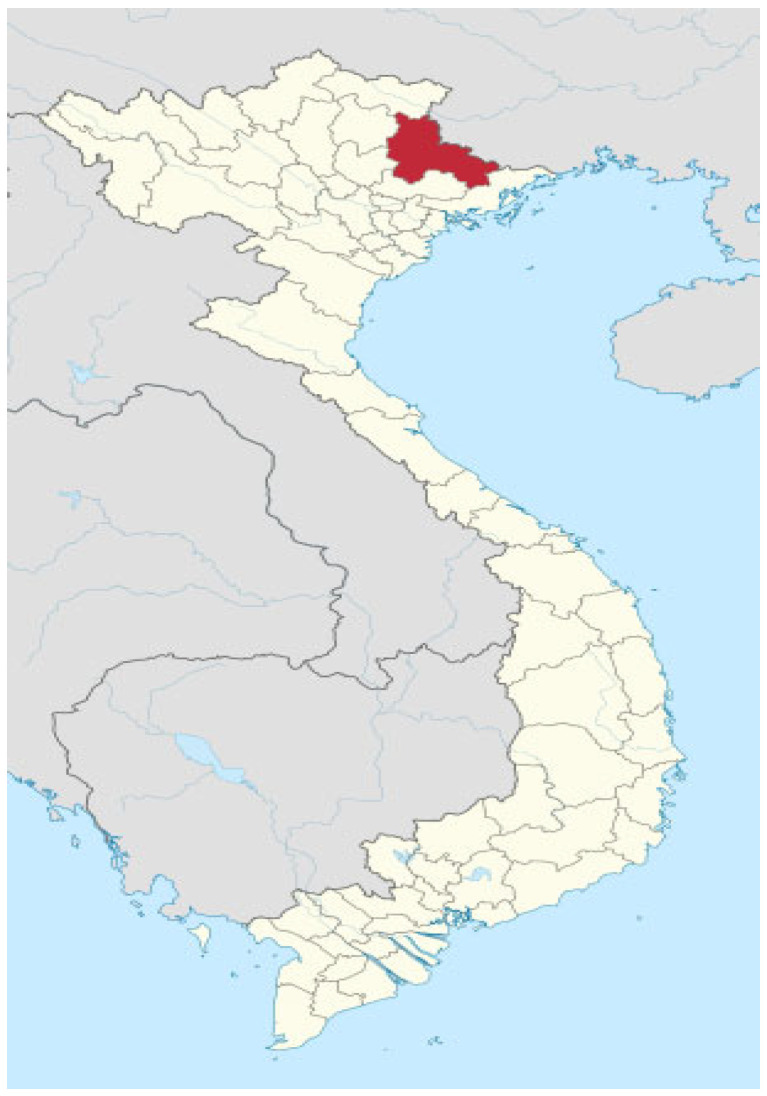
Lang Son Province (Red), Vietnam.

However, as the national burden declines, these aggregate indicators are insufficient for identifying regions with disproportionately high mortality that persist in marginalized areas. Because national-level averages are heavily influenced by success in urban centers, they effectively mask unique epidemiological dynamics in remote border provinces like Lang Son. Sub-national analyses are critical for revealing disparities masked by national data, particularly in mountainous border provinces like Lang Son (Figure 1). Lang Son presents multiple intersecting challenges for TB control and treatment adherence. The province is predominantly rural (80.7%) with rugged highlands that hinder timely diagnosis and follow-up. Nearly 84% of the population belongs to ethnic minority groups, often facing socio-linguistic barriers that limit health literacy and engagement with TB services [10]. Additionally, Lang Son’s role as a major trade gateway [11] through the Huu Nghi border crossing increases population mobility, which can disrupt treatment continuity and facilitate undiagnosed transmission.

While previous research in Vietnam has documented national incidence [12], prevalence [13,14], case notifications [15] and national mortality [16], a significant gap remains regarding longitudinal, sub-national mortality trends. This study addresses this gap by examining provincial-level mortality data, which provides a more detailed understanding of the local epidemiological dynamics that national data often overlook. We focused specifically on pulmonary tuberculosis because it is the transmissible form of the disease responsible for community spread and accounts for the vast majority of TB-related deaths recorded in the Lang Son provincial mortality registry.

The purpose of this study was to (1) analyze temporal trends in pulmonary TB mortality over 12 years from 2005 to 2008 and 2011 to 2018 in Lang Son province and (2) evaluate gender differences in these mortality trends. The study period from 2005 to 2018 is particularly significant as it encompasses a critical implementation phase where Vietnam transitioned from foundational DOTS expansion to the comprehensive WHO End TB Strategy. Evaluating mortality trends during this 12-year window is essential for understanding the long-term impact of early programmatic scale-ups at the provincial level. Furthermore, a clean pre-COVID-19 pandemic baseline, which is essential for assessing the impact of pandemic-related disruptions on TB control and treatment in border regions.

## 2. Materials and Methods

### 2.1. Study Design and Data Source

This population-based retrospective cohort study used data from a population-based mortality registration system from 2005 to 2008 and 2011 to 2018. Pulmonary TB was defined as any death recorded under ICD-10 codes A15–A16 as the underlying cause of death. To ensure a specific and consistent case definition, we excluded cases of disseminated tuberculosis (A19) and other extrapulmonary forms, focusing on pulmonary TB as the primary source of transmission and the high burden of mortality in the province. Each commune health station’s head physician verified causes of death using medical records, treatment cards, or hospital discharge summaries. The unit of analysis was individual deaths, which were aggregated to calculate annual mortality rates. We focused on pulmonary TB, as it represents the primary source of transmission and the highest burden of mortality in this region.

Data were obtained using the A6 mortality system, an official death recording system managed by the Lang Son Center for Disease Control and Prevention (CDC). One of the co-authors was granted permission to access the A6 mortality database. These unique mortality reporting systems were introduced and implemented nationwide in Vietnam in 1992, with mandatory active population-based mortality registration. They have been previously validated, demonstrating good sensitivity, specificity and accuracy for mortality recording [17,18]. The process involved systematic monthly recording of all community deaths at commune health stations (CHSs) using the A6 form. Heads of the health stations were responsible for compiling and verifying the data each month, which was then submitted annually to the Lang Son CDC. This process established a comprehensive mortality database starting in 2005, containing detailed information such as case ID, age, sex, date and place of death, cause of death, and the corresponding ICD-10 code.

### 2.2. Study Setting

The Lang Son population-based mortality registration system annually covers over 226 commune health stations across the province’s 11 cities and districts. These stations report annual data on the populations they serve to the Lang Son CDC, which compiles and manages the records. To ensure accurate and complete records, heads of each commune health station train medical staff in careful monitoring and follow-up of each death case for at least six months. When documentation was incomplete or conflicting, deaths were reviewed using all available sources and classified based on the most consistent clinical and diagnostic information, in accordance with national TB reporting guidelines. Deaths were coded as TB-related only when pulmonary TB was listed as the underlying cause of death and cases with insufficient evidence to confirm TB as the underlying cause were excluded from TB mortality analyses. Despite these efforts, the system may not capture all deaths occurring outside health facilities or provincial borders (e.g., residents referred to central-level hospitals in Hanoi). Therefore, this analysis includes only deaths with well-documented causes of death based on available medical records.

Population data were carefully verified using multiple independent sources, including the provincial statistics department. This study included all pulmonary TB deaths recorded in Lang Son Province between January 2005 and December 2018, totaling 316 cases, representing 0.64% of the total 49,253 all-cause deaths registered in the province. Individuals from China or other provinces who were temporarily living in Lang Son were not included in the analysis to ensure valid population-based mortality rate calculations. The A6 mortality registration system records all deaths occurring within commune boundaries, regardless of residency status, whereas population denominators from Vietnam’s General Statistics Office represent the usual resident population—defined as individuals residing in the province for six months or more or intending to remain [19]. Including deaths among transient individuals without corresponding population-at-risk denominators would inflate mortality rates and misrepresent the true disease burden among the resident population. This approach is consistent with standard demographic methods and previous mortality surveillance studies using the A6 system in Vietnam [17,18].

### 2.3. Data Analysis

Data cleaning, data management, and graphical visualizations were performed using Microsoft Excel, while all statistical analyses were performed using STATA version 15.0 (StatCorp LLC, College Station, TX, USA). Crude annual pulmonary TB mortality rates were calculated by dividing the number of TB deaths in each calendar year by the corresponding mid-year population estimates obtained from the General Statistics Office of Vietnam and expressed per 100,000 population [19].

The crude mortality rate (CMR) was calculated as:

$$\mathrm{CMR}=\left(\frac{\mathrm{Total}\;\mathrm{TB}\;\mathrm{deaths}\;\mathrm{in}\;a\;\mathrm{given}\;\mathrm{year}}{\mathrm{Mid}\text{-}\mathrm{year}\;\mathrm{population}\;\mathrm{of}\;\mathrm{the}\;\mathrm{same}\;\mathrm{year}}\right)\times 100,000$$

Age-standardized mortality rates (ASRs) were estimated using the WHO World Standard Population for 2000–2025 [20]. The proportion of deaths occurring before age 70 was calculated to assess premature mortality burden. This threshold aligns with international frameworks for defining premature mortality, including WHO and United Nations Sustainable Development Goal (SDG) Target 3.4 definitions [21,22], and represents a pragmatic benchmark for distinguishing potentially preventable deaths among the economically productive population from age-related mortality in older age groups [23].

The ASR was calculated as:

$$\mathrm{ASR}=\frac{\sum (r_{i}\times p_{i})}{\sum p_{i}}$$

where $r_{i}$ represents the age-specific mortality rate in age group i, and $p_{i}$ represents the population of age group i in the WHO World Standard Population.

Mortality rate ratios (MRR) and their corresponding 95% confidence intervals (95% CI) were calculated to assess trends in pulmonary TB mortality from 2005 to 2018, adjusting for age groups (0–9, 10–19, 20–29, 30–39, 40–49, 50–59, 60–69, 70–79, 80+) and sex. The year 2005 was used as the reference category for trend comparisons. The mortality rate ratio and 95% confidence interval were estimated for 2006–2008 and 2011–2018. Data on pulmonary TB-related deaths were unavailable for 2009 and 2010 because completed mortality registration forms from all commune health stations were lost, and therefore, data from these two years were excluded from the analysis. Therefore, our analysis covers two periods: 2005–2008 and 2011–2018 for a total of 12 years of observation over a 14-year timeframe. We checked the data for missing values by year and for each commune health station. Only data entries that had both the number of pulmonary TB deaths and the population size for each year and each commune health station were used. These complete records were then combined to create the final dataset for province-wide analysis.

To test temporal trends, Poisson regression models were used with mid-year provincial population as an offset and calendar year as a continuous independent variable. *p*-values from these models were used to assess whether mortality rates changed significantly over time for the overall population and by sex.

## 3. Results

### 3.1. Overall Burden of Pulmonary TB Mortality and Sex-Specific Disparities

Table 1 shows the results of pulmonary TB mortality rates in the province from 2005 to 2008 and 2011 to 2018, classified under ICD codes A15–A16 and stratified by sex. Over the 12-year observation period, 316 pulmonary TB deaths were recorded, representing 0.64% of all deaths in the province during these years (316/49,253). The age-standardized mortality rate (ASR) was 4.0 per 100,000 person-years. Among men, 240 deaths occurred, corresponding to an ASR of 6.9 per 100,000 person-years, while women accounted for 76 deaths, with an ASR of 1.7 per 100,000 person-years. The male-to-female ASR ratio was 4.1, highlighting substantially higher mortality among men. Notably, the majority of TB deaths (69.9%) occurred before age 70.

### 3.2. Temporal Trends in Pulmonary TB Mortality

Table 2 presents annual pulmonary TB mortality from 2005 to 2008 and 2011 to 2018. The overall ASR declined significantly from 6.3 per 100,000 person-years in 2005 to 3.2 in 2018 (*p* = 0.025), representing a 49.2% reduction over the study period. The adjusted mortality rate ratio (MRR) indicated a significant annual decrease (MRR = 0.962; 95% CI: 0.934–0.990; *p* = 0.009).

When stratified by sex, significant downward trends were observed in both men and women. Among men, the ASR decreased from 9.7 per 100,000 person-years in 2005 to 6.2 in 2018, corresponding to an annual reduction of 3.8% (MRR = 0.962; 95% CI: 0.934–0.990; *p* = 0.009). Among women, the ASR declined from 3.4 to 0.8 per 100,000 person-years, corresponding to an annual reduction of 6.1% (MRR = 0.939; 95% CI: 0.891–0.989; *p* = 0.018).

### 3.3. Pulmonary TB Mortality by Geographic Area, District and Sex

Table 3 presents data on pulmonary TB mortality across districts, stratified by levels of urbanization/social status during 2005–2008 and 2011–2018. Mortality rates varied substantially across districts and geographic settings. Higher TB mortality was observed in sub-urbanized and urban areas compared with rural and high-mountain districts. Among sub-urbanized districts, Cao Loc (5.5 per 100,000 person-years) showed the highest mortality rate, followed by Van Quan with a moderately high rate (3.7 per 100,000). Lang Son City, the only urban district, also showed relatively high TB mortality (3.6 per 100,000). In contrast, rural districts (e.g., Huu Lung) had lower mortality rates (3.2 per 100,000), and the lowest rates were observed in high-mountain districts, particularly Binh Gia (1.5 per 100,000). Across all districts, males consistently experienced higher TB mortality than females, with male-to-female rate ratios ranging from approximately two- to fivefold.

### 3.4. Age-Specific Pulmonary TB Mortality Rates by Sex

**Figure 2:**
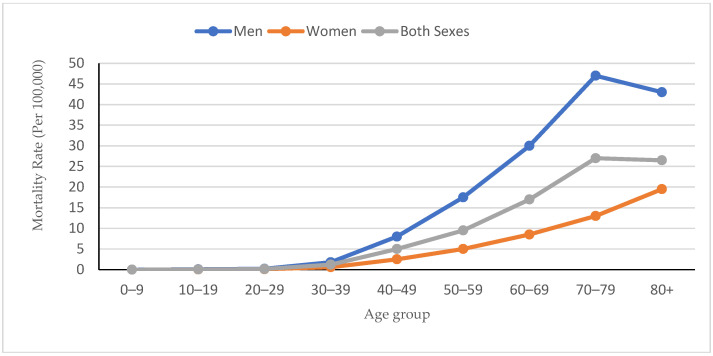
Age and sex-specific mortality rates per 100,000 person-years for 2005–2008 and 2011–2018 (n = 316).

Figure 2 shows age-specific mortality rates per 100,000 person-years, stratified by sex from 2005 to 2008 and 2011 to 2018. Mortality rates increased progressively with age for both men and women, with men exhibiting consistently higher rates across all age cohorts. While mortality remained low in the younger age groups (0–49 years), rates rose substantially after age 50 with the highest mortality observed in the oldest cohorts (70 years and older). As shown in Table 4, men exhibited significantly higher mortality rates, peaking in the 70–79 age group at 48.6 per 100,000, followed by 43.3 in the ≥80 age group and 30.2 in the 60–69 age group. In contrast, women had lower mortality rates overall, with their peak occurring in the ≥80 age group (19.1 per 100,000).

## 4. Discussion

To our knowledge, this is the first population-based analysis of TB mortality trends at the sub-national level in Vietnam, providing empirical evidence of the epidemiological shifts within a specific provincial context. The study identified a substantial decline in pulmonary TB-related mortality in Lang Son Province between the 2005–2008 and 2011–2018 periods. While this downward trend occurred during the post-DOTS expansion era in Vietnam, the ecological design of this study describes the shift in disease burden without attributing these changes to specific programmatic interventions or clinical practices.

The findings offer an essential pre-COVID-19 baseline that characterizes the state of TB mortality through 2018. This longitudinal baseline is a critical reference point for evaluating the subsequent impact of Vietnam’s National Strategic Plan (2021–2025) and for monitoring progress toward the 2030 End TB targets. Furthermore, these data provide the necessary context to determine whether future mortality shifts represent a continuation of earlier trends or a reversal caused by COVID-19 pandemic–related disruptions. Establishing these pre-pandemic trends in a border province is necessary to distinguish long-term epidemiological trajectories from short-term pandemic setbacks, thereby providing a more accurate foundation for future evidence-based interventions.

Despite overall progress, men faced a disproportionately higher TB mortality burden than women. While our data cannot identify the underlying causes, these findings align with previous national surveillance data showing a higher TB prevalence among Vietnamese men compared to women [24]. The finding is consistent with global evidence demonstrating a significantly higher TB burden among men in multiple countries, including China [25], India [26] and Thailand [27]. However, it is important to note that the declining trend observed in women was not statistically significant (*p* > 0.05), indicating that the overall reduction in provincial TB mortality is primarily driven by trends observed in the male population. Due to the ecological design of this study, we cannot determine whether this gender gap reflects biological differences, variations in social risk factors, or differences in healthcare access. Future research incorporating behavioral, social, and healthcare-access variables is needed to better understand the drivers of this disparity. Such studies would help clarify whether the mortality differences reflect true variations in disease burden, differential access to diagnosis and treatment, or other contextual factors not captured in the current study. Importantly, as a single-province study conducted in a specific border region, these findings are context-specific to Lang Son and may not be generalizable to other regions of Vietnam.

Pulmonary TB mortality in Lang Son increased with age, peaking among the oldest cohorts. This age-specific gradient is consistent with the Global Burden of Disease 2021 report and previous studies in Vietnam [28,29,30]. Specifically, the high mortality rates observed in those aged 70 and older identify this demographic as a high-risk group within the provincial mortality profile. The Global Burden of Disease Study 2021 similarly reported that TB mortality rates increase with age, with older adults (≥70 years) accounting for 24.5% of global TB deaths despite representing a smaller proportion of the overall population [28]. While this concentration of deaths in older age groups aligns with the demographic shift toward an aging population in Vietnam, the ecological nature of our data does not allow for an assessment of the underlying clinical or social drivers. The observed trend highlights a demographic disparity in mortality, though further empirical study is required to determine the specific healthcare needs or effective screening strategies for this population.

This study also reveals significant geographic disparities in pulmonary TB mortality across Lang Son Province. Districts characterized by higher levels of urbanization or semi-urbanization exhibited higher mortality rates than some more remote high-mountain districts. The result is consistent with a previous national study in Vietnam showing that those living in an urban center were associated with an increased TB prevalence compared to rural and mountainous areas [30]. While the observed differences may reflect unmeasured contextual factors such as population density or healthcare access, the ecological nature of these data does not allow for causal inferences. These findings highlight significant spatial variations in TB mortality that warrant further investigation through individual-level or service-access research.

Our study excluded temporary residents from China or other provinces to ensure valid population-based mortality rate calculations. However, Lang Son’s role as a major economic gateway through the Huu Nghi border gate, one of the busiest crossings between Vietnam and China with substantial daily traffic, results in significant cross-border movement of people who may not be captured in resident registries [31]. These mobile populations, including traders, seasonal migrants, and other travelers, face unique TB vulnerabilities, such as interrupted treatment and barriers to continuous care, which have been documented among migrant groups in other settings [32,33]. While our findings reflect robust mortality trends for the resident population, they likely underestimate the total TB burden occurring within the province, and cross-border movement may influence local transmission dynamics, potentially sustaining higher mortality in semi-urban and border-adjacent districts. Future research using cross-border surveillance data is needed to capture TB outcomes in these highly mobile populations and provide a more complete picture of the provincial burden.

This study has some limitations. First, reliance on the A6 mortality registration system may have resulted in underreporting or misclassification of TB deaths, particularly those occurring outside health facilities or without complete medical documentation. Deaths recorded in hospital-based registers may have been missed if not reported to commune health stations. However, such misclassification is likely nondifferential over time and therefore unlikely to substantially bias the observed declining mortality trends. Second, the small number of annual deaths limited statistical power to detect sex-specific trends, although the overall decline was statistically significant. Third, the missing TB death data for 2009 and 2010 may affect the overall outcomes; however, the consistent declining trend observed in both observation periods (2005–2008 and 2011–2018) indicates that these gaps likely did not alter the overall trajectory substantially. Fourth, as a single-province study from a border region, generalizability to other Vietnamese provinces may be limited, particularly to non-border or urban settings. Finally, the ecological design precludes assessment of individual-level risk factors for TB mortality. Despite these limitations, this 12-year population-based study provides rare empirical evidence on sub-national TB mortality and establishes a pre-COVID-19 baseline for monitoring future trends as Vietnam advances toward its 2030 End TB targets.

## 5. Conclusions

Between 2005 and 2008 and 2011 and 2018, Lang Son Province experienced a substantial decline in pulmonary TB mortality, falling from 6.3 to 3.2 per 100,000 person-years. However, this decline was primarily driven by trends in the male population, as the declining trend among women was not statistically significant. In addition, a stark disparity persists, with higher mortality rates concentrated among men and older adults (aged ≥70 years). While the missing data for 2009–2010, the ecological design, and the potential underreporting of TB deaths require a cautious interpretation of the continuous trajectory, these findings establish a critical sub-national baseline for monitoring progress toward Vietnam’s 2030 End TB targets and for distinguishing long-term epidemiological trends from COVID-19 pandemic-related disruptions. Further research using individual-level data is needed to investigate the specific drivers of the observed demographic and geographic disparities in this border province.

## Figures and Tables

**Table 1:** Overall and sex-specific pulmonary tuberculosis mortality rates, Lang Son Province, 2005–2008 and 2011–2018.

Sex	Cause	Total	Crude Rate ^&^	ASR ^@^	% <70 (95% CI) ^#^	WHO ^$^
Men	A15–A16	240	5.3	6.9	74.2 (68.2–79.5)	7.7
Women	A15–A16	76	1.7	1.7	56.6 (44.7–67.9)	1.9
Both sexes	A15–A16	316	3.5	4.0	69.9 (64.6–74.8)	4.4

^&^ Crude rate per 100,000 person-years; ^$^ Age-standardized rate per 100,000 person-years using the World Health Organization standard population for 2000–2025 (WHO); ^#^ Proportion of death cases under 70-year-olds. Data cover 12 years of observation (2005–2008 and 2011–2018). Data for 2009–2010 were unavailable due to incomplete mortality registration during a transitional period in the provincial vital statistics system. ^@^ Age-standardized rate per 100,000 person-years using the SEGI World standard population (in the 1960s, ASR). Men-to-women ratio (ASR-WHO) = 4.1 (7.7/1.9).

**Table 2:** Temporal trends in pulmonary TB mortality rates by sex, Lang Son province, 2005–2008 and 2011–2018.

Year ^#^	Cases	Crude Rate ^&^	% <70 (95% CI) ^#^	WHO ^$^	MRR (95% CI) ^$$^	*p*
Both Sexes						
2005	37	5.1	78.4 (65.3–91.5)	6.3	1 (reference)	
2006	33	4.5	63.6 (45.7–81.5)	5.7	0.877 (0.548, 1.402)	0.583
2007	29	3.9	75.9 (60.4–91.4)	5.0	0.767 (0.472, 1.247)	0.285
2008	28	3.8	75.0 (58.9–91.1)	4.6	0.730 (0.447, 1.193)	0.209
2011	30	4.1	60.0 (42.6–77.4)	5.3	0.796 (0.492, 1.288)	0.352
2012	23	3.1	65.2 (45.8–84.6)	3.7	0.599 (0.356, 1.008)	0.054
2013	21	2.8	61.9 (40.9–82.9)	3.6	0.554 (0.324, 0.946)	0.030
2014	25	3.2	80.0 (64.3–95.7)	4.4	0.622 (0.375, 1.034)	0.067
2015	22	2.8	72.7 (54.1–91.3)	3.6	0.544 (0.321, 0.921)	0.024
2016	26	3.3	65.4 (47.2–83.6)	4.3	0.644 (0.390, 1.064)	0.086
2017	21	2.6	52.4 (30.6–74.2)	3.3	0.514 (0.301, 0.877)	0.015
2018	21	2.7	85.7 (70.7–100)	3.2	0.521 (0.305, 0.890)	0.017
Men						
2005	26	7.3	84.6 (70.9–100)	9.7	1 (reference)	
2006	28	7.7	60.7 (42.5–78.9)	11.0	1.059 (0.621, 1.806)	0.834
2007	20	5.5	85.0 (69.3–100)	7.9	0.753 (0.420, 1.348)	0.339
2008	21	5.7	76.2 (58.2–94.2)	7.8	0.779 (0.438, 1.385)	0.395
2011	24	6.6	66.7 (47.9–85.5)	10.5	0.906 (0.520, 1.578)	0.727
2012	16	4.3	75.0 (54.2–95.8)	5.9	0.593 (0.318, 1.105)	0.100
2013	14	3.8	71.4 (47.7–94.9)	5.2	0.525 (0.274, 1.006)	0.052
2014	18	4.6	83.3 (63.5–100)	7.1	0.638 (0.350, 1.163)	0.142
2015	18	4.6	72.2 (53.1–91.3)	7.2	0.633 (0.347, 1.154)	0.136
2016	20	5.1	75.0 (55.7–94.3)	7.4	0.705 (0.394, 1.263)	0.240
2017	17	4.3	58.8 (35.2–82.4)	6.4	0.591 (0.321, 1.090)	0.092
2018	18	4.6	83.3 (63.5–100)	6.2	0.635 (0.348, 1.159)	0.139
Women						
2005	11	3.0	63.6 (35.2–92.0)	3.4	1 (reference)	
2006	5	1.4	80.0 (44.9–100)	1.8	0.447 (0.155, 1.286)	0.135
2007	9	2.4	55.6 (23.0–88.2)	2.6	0.800 (0.332, 1.932)	0.621
2008	7	1.9	71.4 (38.0–100)	2.1	0.614 (0.238, 1.584)	0.313
2011	6	1.6	33.3 (0–71.1)	1.6	0.535 (0.198, 1.447)	0.218
2012	7	1.9	42.9 (6.0–85.8)	1.9	0.613 (0.238, 1.582)	0.312
2013	7	1.9	42.9 (6.0–85.8)	2.2	0.621 (0.241, 1.601)	0.324
2014	7	1.8	71.4 (38.0–100)	2.3	0.586 (0.227, 1.512)	0.269
2015	4	1.0	75.0 (32.7–100)	1.2	0.332 (0.106, 1.044)	0.059
2016	6	1.5	33.3 (0–71.1)	1.8	0.500 (0.185, 1.352)	0.172
2017	4	1.0	25.0 (0–67.4)	1.2	0.329 (0.105, 1.033)	0.057
2018	3	0.8	100 (29–100)	0.8	0.250 (0.070, 0.898)	0.033

^&^ Crude rate per 100,000 person-years; ^$^ Age-standardized rate per 100,000 person-years using the World Health Organization standard population for 2000–2025 (WHO); ^$$^ adjusted for age group (0–9, 10–19, 20–29, 30–39, 40–49, 50–59, 60–69, 70–79, 80+) and sex (if applicable). Mortality rate ratio and 95% confidence interval: MRR (95% CI). ^#^ missing data 2009–2010.

**Table 3:** Pulmonary TB mortality by geographic area, district/city and sex, Lang Son province, 2005–2008 and 2011–2018.

Geographic Area	District, City	Person-Years			Pulmonary TB Death ^a^			Rate per 100,000		
		Total	Male	Female	Total	Male	Female	Total	Male	Female
Sub-urbanization	Cao Loc	884,618	436,138	450,479	49	34	15	5.5	7.8	3.3
Sub-urbanization	Chi Lang	899,923	449,570	451,912	41	27	14	4.6	6.0	3.1
Sub-urbanization	Van Quan	643,603	316,573	328,353	24	18	6	3.7	5.7	1.8
Urban	Lang Son City	1,103,243	539,842	555,709	40	34	6	3.6	6.3	1.1
Rural	Huu Lung	1,452,087	736,710	746,854	47	39	8	3.2	5.3	1.1
Rural	Loc Binh	977,306	487,157	503,072	28	21	7	2.9	4.3	1.4
High Mountain	Dinh Lap	325,513	161,634	164,574	8	5	3	2.5	3.1	1.8
High Mountain	Trang Dinh	702,625	345,972	357,667	16	11	5	2.3	3.2	1.4
High Mountain	Bac Son	821,118	403,751	419,060	17	14	3	2.1	3.5	0.7
High Mountain	Binh Gia	650,566	335,162	316,040	10	7	3	1.5	2.1	0.9

^a^ Pulmonary TB Death is confirmed by ICD-10: A15–A16.

**Table 4:** Age- and sex-specific pulmonary TB mortality rates (per 100,000 person-years), 2005–2008 and 2011–2018.

Age Group (Years)	Men	Women	Both Sexes
0–9	0.0	0.0	0.0
10–19	0.2	0.0	0.1
20–29	0.3	0.0	0.2
30–39	2.3	0.3	1.3
40–49	7.9	1.2	4.5
50–59	17.4	2.6	9.6
60–69	30.2	8.6	17.8
70–79	48.6	12.9	26.9
80+	43.3	19.1	26.5

## Data Availability

The data that support the findings of this study are available on request from the corresponding author.

## Abbreviations

The following abbreviations are used in this manuscript:

ASRs	Age-standardized mortality rates
CDC	Center for Disease Control and Prevention
CHSs	Commune Health Stations
MRR	Mortality rate ratios
TB	Tuberculosis
WHO	World Health Organization
